# Emotional and behavioural difficulties in children and adolescents with hearing impairment: a systematic review and meta-analysis

**DOI:** 10.1007/s00787-015-0697-1

**Published:** 2015-03-11

**Authors:** Jim Stevenson, Jana Kreppner, Hannah Pimperton, Sarah Worsfold, Colin Kennedy

**Affiliations:** 1 Faculty of Social and Human Sciences, University of Southampton, Highfield, Southampton, SO17 1BJ UK; 2 Faculty of Medicine, University of Southampton, Southampton, UK

**Keywords:** Hearing impairment, Emotional difficulties, Behavioural difficulties

## Abstract

The aim of this study is to estimate the extent to which children and adolescents with hearing impairment (HI) show higher rates of emotional and behavioural difficulties compared to normally hearing children. Studies of emotional and behavioural difficulties in children and adolescents were traced from computerized systematic searches supplemented, where appropriate, by studies referenced in previous narrative reviews. Effect sizes (Hedges’ *g*) were calculated for all studies. Meta-analyses were conducted on the weighted effect sizes obtained for studies adopting the Strength and Difficulties Questionnaire (SDQ) and on the unweighted effect sizes for non-SDQ studies. 33 non-SDQ studies were identified in which emotional and behavioural difficulties in children with HI could be compared to normally hearing children. The unweighted average *g* for these studies was 0.36. The meta-analysis of the 12 SDQ studies gave estimated effect sizes of 0.23 (95 % CI 0.07, 0.40), 0.34 (95 % CI 0.19, 0.49) and −0.01 (95 % CI −0.32, 0.13) for Parent, Teacher and Self-ratings of Total Difficulties, respectively. The SDQ sub-scale showing consistent differences across raters between groups with HI and those with normal hearing was Peer Problems. Children and adolescents with HI have scores on emotional and behavioural difficulties measures about a quarter to a third of a standard deviation higher than hearing children. Children and adolescents with HI are in need of support to help their social relationships particularly with their peers.

## Introduction

The mental health of children with hearing impairment (HI) is of potential concern as their social-emotional development may be negatively impacted by difficulties in communication. Co-occurring cognitive and physical impairments are additional risk factors for many children with HI. These mental health risks may also be exacerbated by experiences within families and within the education system [1–3]. For this reason it is important to determine if, and to what extent, their mental health is less good than that of hearing children so that intervention could be targeted at this possibly vulnerable group. Studies on this question have largely adopted questionnaire measures of emotional and behavioural difficulties (EBD) and there is a wide variation between studies in the prevalence of EBD found in children with HI.

A range of mental health problems have been found to be associated with HI including depression, aggression, oppositional defiant disorder and conduct disorder, and less consistently anxiety, somatization, and delinquency [4]. There is an unresolved issue of whether children with HI show hyperactivity and inattention. It has been suggested that children with HI may be prone to show increased rates of hyperactivity and ADHD symptomatology [5], but this is not a consistent finding across studies. Kelly et al. [6] reported an association between HI and ADHD in acquired HI cases only. Van Eldik [7] found that it was only children with HI and low intelligence that showed attention problems. Finally, the association between HI and ADHD reported by Hindley and Kroll [5] was only significant for school-based hyperactivity, and there was no significant effect relative to the general population on parents’ ratings of hyperactivity. Furthermore, some doubt was cast on the generalizability of this school-based finding by the suggestion that the effect may have arisen from the ratings of just one teacher [5].

In a narrative review of studies on EBD in children with HI, estimates varied between 0 and 77 % in the rates of EBD [8]. There are a number of possible reasons for these discrepancies between studies, including differences in the sampling of children with HI (e.g. age, the extent of other associated impairments, and the nature and severity of hearing loss), differences in the measure used and in the informant on behaviour (i.e. parent, teacher or child). Meta-analysis provides a transparent and replicable method for synthesizing the results of such disparate studies, for discerning the overall mean effect and for identifying studies that do not conform to this general pattern, i.e. outliers [9].

To date there has been no attempt to give a quantitative assessment of the magnitude of differences in the rates of EBD in general, or on specific types of behaviour difficulties, in children with and without HI. This paper will present the results of two quantitative reviews that we have undertaken of studies on EBD in children with HI. One is of studies using a variety of measures of behaviour difficulties. The second is of studies using the Strength and Difficulties Questionnaire (SDQ) [10]. We have analysed separately those studies that did or did not use the SDQ because the non-SDQ studies have varied widely in the instrument used, which may or may not have included subscales. By contrast the SDQ studies, being all based on the same measure, are more homogeneous and provide well-validated sub-scale scores, with equivalent ratings based on parent, teacher and self-report which can be analysed separately but in parallel. The adoption of this measure in a number of recent investigations allows a more definitive appraisal of not only just the overall extent of EBD in children with HI but also of the type of difficulties shown and whether these are equally apparent to parents and to teachers. There are, in addition, a small number of studies using self-ratings on the SDQ by older children and adolescents.

The comparison of the results of these two separate analyses has to be made circumspectly. There are a wide variety of measures used in the non-SDQ review and pooling of results from disparate measures must be treated with caution. The majority of these non-SDQ measures are nevertheless widely adopted in studies of child behaviour and have been well validated.

## Method

### Studies not using SDQ

#### Non-SDQ study inclusion

These were studies of EBD in children and adolescents with HI which did not use the SDQ as a measure. For inclusion, the studies need to provide information from validated questionnaires or interviews on the prevalence of EBD or mean problem scores in children with HI. In addition, data from a control group or norm data from population samples should have been provided. If such comparison data were not provided, studies were still included if such norm data could be obtained subsequently from elsewhere.

#### Non-SDQ study retrieval

The following databases were searched for studies published between 1970 and June 2014: Science Citation Index Expanded (1970–present), Social Sciences Citation Index (1970–present), Arts & Humanities Citation Index (1975–present), Conference Proceedings Citation Index—Science (1990–present), Conference Proceedings Citation Index—Social Science & Humanities (1990–present), MEDLINE, PUBMED and PSYCHInfo. The search terms were (1) child* or adolescent* AND (2) deaf* or hearing or PCHI or cochlear implant* or hearing aid AND (3) behaviour problems or mental health.

To ensure maximum coverage of this disparate literature, citations in two authoritative earlier reviews [8, 11] and a recently published systematic review [4] were also checked.

#### Non-SDQ study selection

**Fig. 1:**
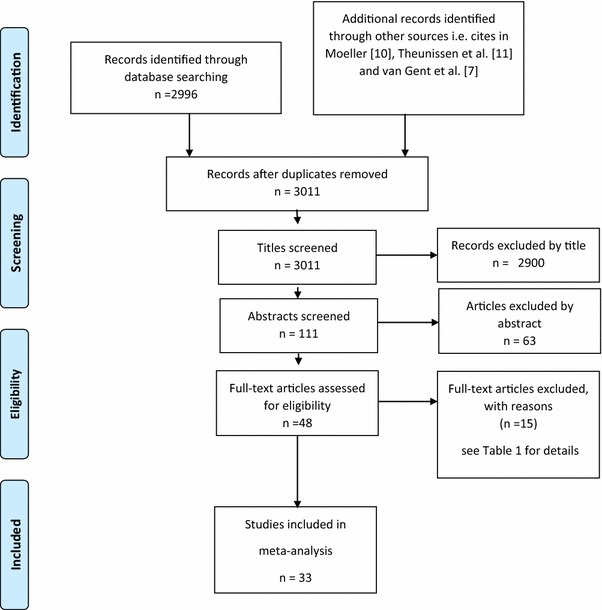
PRISMA flow chart of selection of non-SDQ studies

The search identified 48 papers that possibly could have been included but 15 of these studies did not meet the inclusion criteria. These papers and the reason for their exclusion are given in Table 1. From these various sources, there were therefore a total of 33 papers on non-SDQ EBD measures that met the inclusion criteria and details of these papers are given in Table 2. A summary of the selection of the non-SDQ studies is given in Fig. 1 in the form of a PRISMA flow chart [71].

**Table 1:** Non-SDQ studies identified in the systematic search but excluded from the meta-analysis

Study	Reason for exclusion
Aplin [12]	Data not able to be compared with hearing controls
Aplin [13]	Data not able to be compared with hearing controls
Barker et al. [14]	Data not able to be compared with hearing controls
Bat-Chava and Deignan [15]	Data not able to be compared with hearing controls
Bat-Chava, Martin and Kosciw [16]	Data not able to be compared with hearing controls
Bizjak [17]	Behaviour measured with instrument of uncertain equivalence to those used in other studies
Freeman, Malkin, and Hastings [18]	Data not able to be compared with hearing controls
Gallaudet research institute [19]	Data not able to be compared with hearing controls
Hindley et al. [6]	Data not able to be compared with hearing controls
Keilman, Limberger, and Mann [20]	Data not able to be compared with hearing controls
Kent [21]	Behaviour measured with instrument of uncertain equivalence to those used in other studies
King, Mulhall, and Gullone [22]	Behaviour measured with instrument of uncertain equivalence to those used in other studies
Kouwenberg et al. [23]	Behaviour measured with instrument of uncertain equivalence to those used in other studies
Maes and Grietens [24]	Data not able to be compared with hearing controls
Polat [25]	Data not able to be compared with hearing controls

**Table 2:** Characteristics of studies on EBD of children and adolescents with HI not using the SDQ

	Country	Nature of HI dB loss % Cochlear implant (CI)	Age in years	Measure	% With mental health problems or Mean and SD on mental health measure		Hedges’*g*
					Hearing impaired group	Control or norm group	
Anderssen et al. [26]	Sweden	70 % severe or mild hearing loss in both ears 30 % severe or mild hearing loss in one ear	7–12	Rutter Parent Scale^e^			
				Internalizing	Mean = 1.95	Mean = 1.84	0.16
					SD = 0.88	SD = 0.59	
					*N* = 57	*N* = 187	
				Externalizing	Mean = 1.73	Mean = 1.59	0.24
					SD = 0.75	SD = 0.52	
					*N* = 57	*N* = 187	
				Rutter Teacher Scale^b^			
				Internalizing	Mean = 2.04	Mean = 1.81	0.28
					SD = 0.89	SD = 0.80	
					*N* = 48	*N* = 208	
				Externalizing	Mean = 1.41	Mean = 1.40	0.01
					SD = 0.63	SD = 0.70	
					*N* = 48	*N* = 208	
Arnold and Atkins [27]	England	Mean 66.7 dB loss	4–11	Bristol Social Adjustment Guide^a^	44 % (*n* = 10/23)	30 % (*n* = 7/23)	0.30
				Rutter Teacher Scale^b^	0 %	0 %	–
Brubaker and Szakowski [28]	USA	8 % 30–44 dB 13 % 45–59 dB 18 % 60–79 dB 61 % 80 + dB	3–18	Eyberg Child Behavior Inventory			
				Intensity scale	Mean = 104.15	Mean = 91.43	0.52
					SD = 25.86	SD = 22.86	
					*N* = 39	*N* = 37	
				Problem scale	Mean = 8.05	Mean = 6.39	0.27
					SD = 6.54	SD = 5.90	
					*N* = 39	*N* = 37	
Cornes et al. [29]	Australia	15 % severe 85 % profound	11–18	Youth Self Report^n^			
				Internalizing	17.9 % (*n* = 5/28)	19.6 % (*n* = 250/1273)	−0.06
				Externalizing	25.0 % (*n* = 7/21)	16.4 % (*n* = 209/1273)	0.29
Davis et al. [30]	USA	40 % < 44 dB 37.5 % 45–60 dB 22.5 % > 61 dB	5–18	Child Behavior Checklist^c^			
				Internalizing	Mean = 53.0	Mean = 50.0^h^	0.30
					SD = 10.1	SD = 10	
					*N* = 40	*N* = 300	
				Externalizing	Mean = 54.2	Mean = 50.0^h^	0.42
					SD = 10.4	SD = 10	
					*N* = 40	*N* = 300	
Edwards et al. [31]	England	Profound pre-CI	2–5	CBCL^g^			
				Internalizing	Mean = 50.5	Mean = 50.0^h^	0.05
					SD = 7.2	SD = 10.0	
					*N* = 17	*N* = 300	
				Externalizing	Mean = 51.2	Mean = 50.0^h^	0.12
					SD = 8.3	SD = 10.0	
					*N* = 17	*N* = 300	
Fundudis et al. [32]	England	Deaf not further specified	7–10	Rutter Teacher Scale^b^	44 % (*n* = 24/54)^b^	18 % (*n* = 18/102)^i^	0.72
Furstenberg and Doyal [33]	USA	80 % Serious or profound hearing loss in both ears	11–21	Teacher Report Form^d^	Average across grades		
				Internalizing	Mean = 53.71	Mean = 50.0^j^	0.38
					SD = 8.73	SD = 10	
					*N* = 63	*N* = 300	
				Externalizing	Mean = 53.16	Mean = 50.0^j^	0.33
					SD = 6.95	SD = 10	
					*N* = 63	*N* = 300	
Hindley and Kroll [5]	England	>40 dB	11–16	Rutter Parent Scale^e^ Rutter Teacher Scale^b^	Hyperactive home	Hyperactive home	−0.15
					9.9 % (*n* = 8/81)	12.7 % (*n* = 63/498)	
					Hyperactive school	Hyperactive school	0.48
					16.0 % (*n* = 13/81)	7.2 % (*n* = 36/498)	
					Hyperactive pervasive	Hyperactive pervasive	0.37
					8.6 % (*n* = 7/81)	4.6 % (*n* = 23/498)	
Kammerer [34]	Germany	>20 dB	10–13	Rutter Teacher Scale^b^	54 % (*n* = 99/183)^b^	16 %^i^	1.00
Kelly et al. [6]	USA	89 % Severe or greater loss	4–21	Conners’ Parent Rating Scale^f^	Females	Females	
				Impulsive-hyperactive	Mean = 0.77	Mean = 0.83	−0.09
					SD = 0.72	SD = 0.61	
					*N* = 97	*N* = 238	
				Impulsive-hyperactive	Males	Males	
					Mean = 0.66	Mean = 0.89	−0.39
					SD = 0.65	SD = 0.59	
					*N* = 115	*N* = 291	
				Hyperactivity	Females	Females	
					Mean = 0.52	Mean = 0.55	−0.07
					SD = 0.55	SD = 0.39	
					*N* = 97	*N* = 238	
				Hyperactivity	Males	Males	
					Mean = 0.52	Mean = 0.65	−0.29
					SD = 0.49	SD = 0.44	
					*N* = 115	*N* = 291	
Konuk et al. [35]	Turkey	3 % 56–70 db 8 % 71–90 db 89 % > 91 db	6–18	Child Behavior Checklist^c^			
				Internalizing	Mean = 57.84	Mean = 52.11	0.50
					SD = 11.78	SD = 10.74	
					*N* = 72	*N* = 45	
				Externalizing	Mean = 51.98	Mean = 50.11	0.15
					SD = 12.02	SD = 12.24	
					*N* = 72	*N* = 45	
Kouwenberg et al. [36]	The Netherlands	>40 dB 37 % CI	8–15	Child Depression Inventory^o^	Mean = 1.39	Mean = 1.33	0.29
					SD = 0.21	SD = 0.20	
					*N* = 78	*N* = 130	
Li and Prevatt [37]	China	Deaf not further specified	8–19	Revised Children’s Manifest Anxiety Scale^s^	Females	Females	
					Mean = 14.30	Mean = 10.61	0.71
					SD = 5.10	SD = 5.08	
					*N* = 30	*N* = 34	
					Males	Males	
					Mean = 15.55	Mean = 11.18	1.08
					SD = 3.36	SD = 4.50	
					*N* = 31	*N* = 30	
Mitchell and Quittner [38]	USA	53 % 70–100 dB 47 % > 100 dB	6–14	Child Behavior Checklist^c^	Mean = 58.0	Mean = 50.0^h^	0.79
					SD = 10.5	SD = 10.0	
					*N* = 39	*N* = 300	
				Teacher Report Form^d^	Mean = 56.8	Mean = 50.0^j^	0.71
					SD = 5.2	SD = 10.0	
					*N* = 39	*N* = 300	
Prior et al. [39]	Australia	All aided 30–110 dB	2–5	Teacher rating PBQ			
				Total score	*t* = 3.72, df = 50		1.05^q^
Quittner et al. [40]	USA	Severe to profound	Under 5	CBCL^n^			
				Total score Based on full information Imputed values	Mean = 24.81	Mean = 18.73	0.27
					SD = 21.52	SD = 14.29	
					*N* = 181	*N* = 92	
Remine and Brown [41]	Australia	Deaf not further specified	6–18	Child Behavior Checklist^c^			
				Internalizing	16.9 % (11/65)	13.3 % (433/3255)	0.16
				Externalizing	13.8 % (9/65)	12.7 % (413/3255)	0.06
				Youth Self-Report^n^			
				Internalizing	11.4 % (4/35)	16.4 %(209/1273)	−0.23
				Externalizing	8.6 %(3/35)	19.6 %(249/1273)	−0.53
Rutter, Graham, and Yule [42]	England	At least 40 dB loss	5–14	Interview and questionnaires	15 % (*n* = 2/13)^b^	7 % (*n* = 153/2189)^i^	0.49
Sahli, Arslan, and Belgin [43]	Turkey	100 % with CI	6–18	Rosenberg Self-Esteem Scale^t^			
				Depressive emotioning	Mean = 3.02	Mean = 2.30	0.67
					SD = 1.68	SD = 0.53	
					*N* = 30	*N* = 60	
Sinkkonen [44]	Finland	Deaf not further specified	6–16	Rutter Teacher Scale^b^	21 % (*n* = 62/294)^b^	16 % (*n* = 37/234)^i^	0.19
Tharpe et al. [45]	USA	At least 80 dB loss 32 % with CI	8–14	Child Behavior Checklist^c^	Mean = 47.07^k^	Mean = 37.20	1.11
					SD = 6.80	SD = 11.25	
					*N* = 18	*N* = 10	
				Teacher Report Form^d^	Mean = 46.48^k^	Mean = 46.00	0.05
					SD = 7.56	SD = 11.25	
					*N* = 17	*N* = 8	
Theunissen et al. [46]	The Netherlands	24 % 40–60 dB loss 28 % 61–90 dB loss 34 % > 90 dB loss 14 % Not known 33 % CI	8–16	Child Depression Inventory^o^	Mean = 1.38	Mean = 1.32	0.30
					SD = 0.21	SD = 0.19	
					*N* = 83	*N* = 117	
Theunissen et al. [47]	The Netherlands	26 % 40–60 dB loss 21 % 61–90 dB loss 53 % > 90 dB loss 14 % Not known	9–16	Child symptom inventories^p^			
				Generalized anxiety disorder	Mean = 1.50	Mean = 1.36	0.35
					SD = 0.46	SD = 0.35	
					*N* = 72	*N* = 98	
Theunissen et al. [48]	The Netherlands	24 % 40–60 dB loss 21 % 61–90 dB loss 49 % > 90 dB loss 7 % Not known	8–16	Child symptom inventories^p^			
				ADHD	*t* = 2.84 df = 192		0.41^q^
				ODD	*t* = 2.65 df = 192		0.38^q^
				CD	*t* = 3.30 df = 192		0.48^q^
Topol et al. [49]	USA	40 % Unilateral or < 40 dB loss 60 % > 40 dB loss	1.5–2	Child Behavior Checklist 1.5–5^l^	Mean = 45.9	Mean = 43.5	0.26
					SD = 5.2	SD = 10.3	
					*N* = 30	*N* = 91	
Van Eldik [50]	The Netherlands	+80 dB	6–11	CBCL^c^			
				Total score	*t* = 2.10, df = 493		0.19^q^
Van Eldik et al. [7]	The Netherlands	>90 dB	4–18	Child Behavior Checklist^c^	41 % (*n* = 98/238)	16 % (*n* = 331/2068)	0.72
Van Eldik [51]	The Netherlands	>25 dB	11–18	Youth Self Report^g^	37 % (*n* = 75/202)	17 % (*n* = 173/1016)	0.58
Van Gent et al. [8]	The Netherlands	19 % 73–95 dB 81 % > 95 dB	13–21	Child Behavior Checklist^c^	28 % (*n* = 16/58)	16 %	0.38
				Teacher Report Form^d^	32 % (*n* = 22/68)	17 %	0.47
Vostanis et al. [2]	England	Severe to profound	2–18	Child Behavior Checklist^c^	40 % (*n* = 29/73)	8 %^m^	1.11
Wake et al. [52]	Australia	22 % 20–40 dB 31 % 41–60 dB 17 % 61–80 dB 29 % > 80 dB	7–8	Child Behavior Checklist^c^	36 % (*n* = 36/77)	12 % (*n* = 24/198)	1.02
				Teacher Report Form^d^	20 % (*n* = 16/80)	8 % (*n* = 7/86)	0.58
Watt and Davis [53]	USA	>90 dB loss	Mean age = 13.7 years	Beck Depression Inventory^r^	Mean = 10.52	Mean = 6.62	0.66
					SD = 5.59	SD = 6.13	
					*N* = 50	*N* = 30	

^a^Stott [54]

^b^Rutter [55]

^c^Achenbach and Edelbrock [56]

^d^Achenbach and Edelbrock [57]

^e^Rutter, Tizard and Whitmore [58]

^f^Goyette, Conners and Ulrich [59]

^g^Achenbach [60]

^h^Based on original standardization of CBCL

^i^Taken from van Gent et al. [8]

^j^Based on original standardization of TRF

^k^Average of cochlear implant and hearing aid groups

^l^Achenbach [61]

^m^Estimated from normal curve distribution percentage with T score greater than 64

^n^Achenbach and Rescorla [62]

^o^Kovacs [63]

^p^Gadow and Sprakin [64]

^q^ $$d = \frac{2t}{{\sqrt {\text{df}} }}$$

^r^Beck et al. [65]

^s^Reynolds and Richmond [66]

^t^Rosenberg [67]

^u^Eyberg and Ross [68]

^v^Behar and Springfield [69]

^w^Sawyer et al. [70]

### Studies using SDQ

#### SDQ study inclusion

To be included the studies had to provide summary statistics on either a continuous scale (means and SD) or in categorical form (percentage with abnormal scores) for children or adolescents with HI on any of the Parent, Teacher or Self-rated versions of the SDQ. Each of these three versions of the SDQ gives an overall Total Difficulties score and five sub-scale scores for Emotional Symptoms, Conduct Problems, Hyperactivity, Peer Problems and Prosocial Behaviour. A positive effect size indicates lower Prosocial scores for children with HI than hearing controls but higher scores for children with HI on the other SDQ sub-scales and Total Difficulties. These scores could be compared either with a normally hearing control group or with population norms. The search was limited to published papers to ensure a level of methodological adequacy and rigour amongst those included and to avoid the inevitable problems with securing access to a full set of unpublished studies and the bias that this would introduce [72].

#### SDQ study retrieval

The following databases were searched for studies published between 1995 (when the SDQ was first published) and June 2014: Science Citation Index Expanded, Social Sciences Citation Index, Arts & Humanities Citation Index, Conference Proceedings Citation Index—Science, Conference Proceedings Citation Index—Social Science & Humanities, MEDLINE, PUBMED and PSYCHInfo. The search terms were (1) measure: SDQ or Strengths and Difficulties Questionnaire, AND (2) Participants: deaf* or Hearing or PCHI or Cochlear implant* or Hearing aid.

#### SDQ study selection

**Fig. 2:**
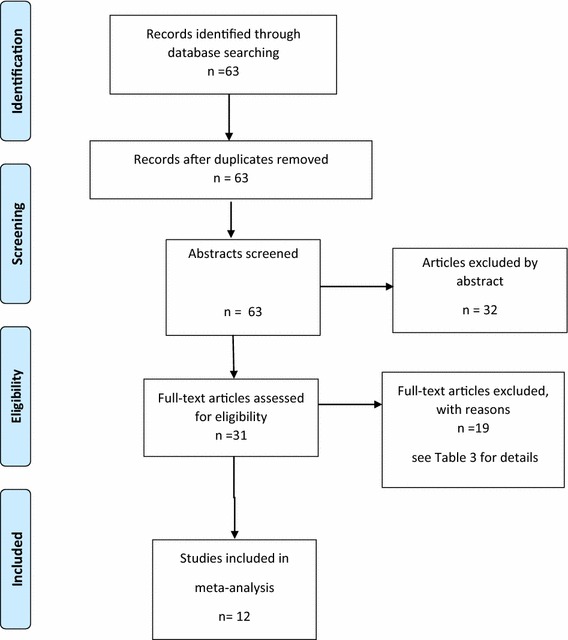
PRISMA flow chart of selection of SDQ studies

The search identified 31 possible studies for inclusion. Of these 19 failed to meet the criteria above for study inclusion. The reasons for exclusion of these 19 studies are summarized in Table 3. The details of the 12 studies included in the analysis are presented in Table 4. The study selection was made independently by two of the authors (JS and HP). In the case of disagreements adjudication was made by a third author (CK). There were two studies where there was uncertainty over their inclusion. One was a study of children with Usher syndrome with complex disabilities in addition to hearing loss [73]. An additional issue with this paper was that SDQ scores were not reported for six children with diagnosed “mental or behavioural disorder”. The second paper [74] studied children with HI associated with a persistent or recurrent history of middle ear disease. It was decided to include both these studies but to conduct a sensitivity analysis to determine if their exclusion modified the results materially. The data extraction was undertaken by JS and the accuracy of data extraction from the papers was determined by a second author (HP) independently checking the summary statistics used to derive the effect sizes for each study. A summary of the selection process for the SDQ studies is given in Fig. 2.

**Table 3:** SDQ studies identified in the systematic search but excluded from the meta-analysis

Study	Reason for exclusion
Fellinger et al. [75]	Sub-set of participants reported in Fellinger et al. [99]
Fellinger et al. [76]	Same participants as in Fellinger et al. [99]
Fellinger and Holzinger [77]	Not peer reviewed
Garg et al. [78]	No report of findings by hearing loss, although neurofibromatosis type 2 is associated with hearing loss
Gurney et al. [79]	Did not use the SDQ
Hintermair [80]	Duplicate of data in Hintermair [101]
Hintermair [81]	Did not provide means and SDs
Hutchison and Gordon [82]	Not only children with HI
Ketelaar et al. [83]	Non-standard use of sub-set of SDQ items
Marret et al. [84]	Not only children with HI
McCormack et al. [85]	Not only children with HI
Moller [86]	Adults with complex disabilities
Muigg, Nekahm-Heis, and Juen [87]	Did not provide means and SDs
Rieffe, Ketelaar, and Wiefferink [88]	Not on children with HI
Saigal et al. [89]	Not just children with HI
St Clair et al. [90]	Not just children with HI
Stevenson et al. [1]	Same sample as Stevenson et al. [107]
Sumpter et al. [91]	Not just children with HI
Watson and Brown [92]	No new data—editorial

**Table 4:** Characteristics of studies on EBD in children and adolescents with HI using the SDQ

Study	Country	No. of hearing impaired	Nature of HI % Cochlear implant (CI)	Age HI in years	SDQ ratings^a^	Hearing controls	Other comparison used
Anmyr et al. [93]	Sweden	22	41-69 dB loss 5 % 70-94 dB loss 14 % >95 dB loss 81 % CI 100 %	9–15	P, T, S	None	Meltzer et al. [94]
Cornes and Brown [95]	Australia	54	>90 dB CI 18.5 %	11–18	P, T, S	None	Used Goodman [96] UK norms
Dammeyer [97]	Denmark	334	>80 dB loss 36 %, <80 dB loss 36 %, CI 28 %	6–19	T	None	Used Smedje et al. [98]. Swedish hearing sample
Dammeyer [73]	Denmark	17	Hearing loss confirmed by standard hearing tests completed by a clinical audiologist	3–17	T	None	Used Smedje et al. [98] Swedish hearing sample
Fellinger et al. [99]	Austria	99	Bilateral hearing loss of at least 40 dB CI 20.9 %	6:5–16	P, T	None	Used norms from Germany Worner, et al. [100] and British SDQ standardization samples Meltzer et al. [94]
Hintermair [101]	Germany	213	< 70 dB 38 % 70–90 dB 35 % > 90 dB 27 % CI 23.5 %	4–12	P	None	Used existing German norms for the SDQ, Woerner et al. [100]
Hogan et al. [102]	Australia	Cohort B 26	“Hearing problems” reported by parents	Cohort B 5.5 years^b^	P	Cohort B Approx. 4000 normal-hearing children	Not applicable
		Cohort K 93		Cohort K 7.5 years^b^	P	Cohort K Approx. 4000 normal-hearing children	
Huber and Kipman [103]	Austria	35	“Profound” bilateral hearing loss CI 100 %	12–17	P, T, S	212 normal-hearing adolescent peers (mean age 15.0, ranging from 12.3 to 17.9 years)	Not applicable
Mejstad et al. [104]	Sweden	111	Children who had been prescribed hearing aids CI 3.3 %	11–18	P, T, S^c^	None	The scale means found in comparable Nordic countries such as Norway (Van Roy et al. [105]) and Finland (Koskelainen et al. [106])
Stevenson et al. [107]^c^	UK	107	Moderate 40–69 dB loss 54 % Severe 70–94 dB loss 24 % Profound > 95 dB loss 22 % CI 13.6 %	5:5–11:8	P, T^d^	Comparison group of 63 children with normal hearing aged 6:4 to 9:10, born at the same hospitals as those with PCHI	Not applicable
Timmerman et al. [74]	The Netherlands	160	Children with a disease history of persistent or recurrent middle ear disease and suffering from either upper respiratory tract infections (URTI) and/or otitis media with effusion OME on the day of assessment The average bilateral hearing level found was 20.0 dB (S.D. = 11.5 dB, range 1.3–49.4 dB) CI 0 %	4–7	P	None	A community sample of US children participating in the National Health Interview Survey (NHIS) conducted by the National Centre for Health Statistics, was used for comparison (Simpson et al. [108])
Vetter et al. [109]	Germany	57	Degree of loss Mild 9 % Moderate 14 % High 40 % Residual hearing 37 % NB dB levels not specified CI 33 %	6:11–12:7	T^c^	None	None

^a^ *P* parent, *T* teacher, *S* self

^b^This is a longitudinal study with behaviour measured at various waves. The effect sizes were calculated using the average of the adjusted odds ratios across these waves and these ages are the mean of the age ranges concerned

^c^Means and SDs were calculated for all participants pooled together

^d^Means and SDs were calculated by the authors for all participants using the raw data

#### Data for normally hearing comparison groups

The studies may have no controls, their own control group or use a large general population sample for comparison purposes. There is a range of such general population samples that can provide norms for the SDQ. Different studies use different samples for normative comparisons. The use of these general population samples for comparison creates a severe distortion to the standard error of the Hedges’ *g* estimate. The sample sizes are of the order of 100 times the size of that for the studies using control groups. The random effects meta-analysis consequently gives these studies a much greater weight than those using their own controls. To overcome this distortion and to allow the inclusion of studies presenting no comparison data, the effect sizes for the impact of HI on SDQ scores were calculated using a common population sample to provide comparison data for all the studies. The norms provided by Meltzer et al. [94] were selected as these were based on a large British general population sample with norms available for parents (*N* = 10,298) and teachers (*N* = 8208) and for self-ratings for older children (*N* = 4228). For this sample norms are provided for two age ranges (5–10 years and 11–15 years). This therefore allows a limited adjustment for age effects to be achieved. For each study, the mean and SD for the age group norms closest in age to the hearing impaired sample was used for comparison. For those studies reporting only the percentage extreme scorers in the hearing impaired group, the appropriate percentage in the age-matched normative sample was used. These comparisons between children with HI and the general population sample could be made for Parent, Teacher and Self-ratings on the Total Difficulties score, for each of four problem sub-scales and for the Prosocial sub-scale.

### Calculating effect sizes

For both SDQ and non-SDQ studies, the effect size used was Hedges’ *g* which provides a standardized mean difference in scores between children with HI and those with normal hearing as follows:

$${\text{Hedges' }}\,{\it g} \, = \frac{{{\text{Mean}}_{\text{HI}} - {\text{Mean}}_{\text{Controls}} }}{{{\text{SD}}_{\text{Pooled}} }} \left[ {1 - \frac{3}{4N - 9}} \right]$$

where *N* = *N*_HI_ + *N*_Controls_ and

$${\text{SD}}_{\text{Pooled}} = \sqrt {\frac{{\left( {N_{\text{HI}} - 1} \right){\text{SD}}_{\text{HI}}^{2} + \left( {N_{\text{Controls}} - 1} \right){\text{SD}}_{\text{Controls}}^{2} }}{N - 2}} .$$

For studies using a categorical scoring, the log odds ratio was calculated and converted to d and then to g using the methods on p. 47 and p. 2 [9]. In all cases, a positive effect size indicates a higher EBD score for children with HI compared to normally hearing children.

The 32 studies not using the SDQ applied a range of methods of measuring behaviour in children with HI (see Table 2). In addition, these studies used various sources for data on the mental health of normally hearing children, i.e. study-specific control groups and population norms. A meta-analysis usually involves averaging the Hedges’ *g* using a weighting procedure that reflects the confidence in the value of *g*—studies with a smaller standard error, usually associated with larger *N*, get greater weight. We did not do this for non-SDQ studies because of concerns over the heterogeneity in the range of measures used in the studies, the variation in control and norm groups used as comparators and because the effect size was based on means in some cases and percentage of extreme scorers in others. Instead the unweighted average value of Hedges’ *g* is reported (see [110] for a discussion of this approach). In addition to the average effect size for overall behaviour difficulties for these non-SDQ studies, the average effect sizes for internalizing and externalizing behaviours are also presented. The parent-rated child behavior checklist (CBCL) [56] total problem scores were the non-SDQ measure most frequently reported and an average effect size was calculated for these studies also.

In the case of SDQ studies, the above considerations were not relevant and weighted Hedges’ *g* was used to provide a standardized mean difference between children with and without HI for each study. These values were used to undertake a meta-analysis using the *metan* command in Stata [111]. The estimate of the overall effect size was obtained using a random effects model, as the effect sizes in these studies were thought unlikely to be functionally equivalent given that different samples were being studied by different research groups in different countries. For the SDQ studies, a test was made to examine whether the magnitude of differences in behaviour between children with HI and controls changed with age. Meta-regressions were conducted using the *metareg* command in Stata [112] with Hedges’ *g* as the dependent and age the independent variable.

To provide “a like for like” comparison between SDQ and non-SDQ studies, we also report the unweighted Hedges’ *g* for SDQ studies.

## Results

### Studies not using SDQ

The 33 non-SDQ studies provided 57 estimates for the standardized mean difference (*g*) of EBD scores for children with HI and hearing controls or a norm group (see Table 2). The unweighted mean effect size was 0.36 (95 % CI 0.26, 0.46; range −0.53 to 1.11). There were 31 estimates based on Parent reports of behaviour with a mean value of 0.34 (95 %CI 0.21, 0.47; range −0.39 to 1.11). The 17 Teacher rating-based estimates gave a higher mean value of 0.46 (95 % CI 0.28, 0.64; range 0.01 to 1.05). These estimates were a mix of effect sizes derived from continuous and categorical measures of EBD. The mean effect size for the 21 categorical measures was slightly higher (0.38, 95 % CI 0.21, 0.54; range −0.39 to 1.11) than that for the 36 continuous indicators (0.35, 95 % CI 0.23, 0.47; range −0.39 to 1.11). When estimates for internalizing and externalizing symptoms were available, a higher mean value of g was obtained for internalizing symptoms (0.35, 95 % CI 0.19, 0.51; range −0.23 to 1.08) than for externalizing behaviours (0.12, 95 % CI −0.03, 0.26; range −0.53 to 0.48). The highest effect size was obtained for total behaviour scores (0.58, 95 % CI 0.44, 0.72; range 0.05 to 1.11). Of these effect sizes for total behaviour problems five were obtained from continuous scores on the CBCL rated by parents and provided an effect size of 0.52 (95 % CI 0.13, 0.92). There are four studies in Table 2 that were not published in peer-reviewed journals. When these were excluded the unweighted mean effect size was reduced slightly to 0.34 (95 % CI 0.24, 0.45; range −0.53 to 1.11).

### Studies using SDQ

There were 12 SDQ studies which provided 10 Parent, 9 Teacher and 4 Self-rated estimates of effect size for Total Difficulties. There were 10, 6 and 4 estimates available for the SDQ sub-scales for Parents, Teacher and Self-rated scores, respectively. One study provided data on two cohorts each of which had SDQ assessment by parents on multiple occasions [102]. The average percentage of high scorers across the assessment’s multiple pre-specified time points was entered into the meta-analysis separately for each cohort.

The random effects model estimates of effect size are presented in Table 5. There is a significant effect size for Parent (0.23) and Teacher (0.34) ratings of Total Difficulties but not for Self-rated scores (−0.01), respectively. The heterogeneity indices based on *χ* ^2^ are significant for Parent and Teacher ratings. The high values of *I* ^2^ suggest that there are systematic rather than random differences between the estimates from the studies. A larger set of studies would be needed to explore the reason for this heterogeneity using meta-regression.

**Table 5:** Effect sizes (Hedge’s *g*) for SDQ sub-scales rated by parents, teachers and self using random effects estimates for the studies overall and for studies with continuous measures

	No. of studies	Overall				Overall heterogeneity				Continuous only				Continuous heterogeneity				M^a^
		*g*	95 % CI	*Z*	*P*	*χ* ^2^	Df	*P*	*I* ^2^	*g*	95 % CI	*Z*	*P*	*χ* ^2^	Df	*P*	*I* ^2^	*g*
Total difficulties																		
Parent	10	0.23	0.07, 0.40	2.77	0.006	42.27	9	0.001	78	0.20	−0.03, 0.43	1.75	0.080	40.77	5	0.001	88	0.20
Teacher	9	0.34	0.19, 0.49	4.39	0.001	28.08	8	0.001	72	0.31	0.15, 0.46	3.91	0.001	12.50	5	0.001	60	0.31
Self-rated	4	−0.01	−0.32, 0.13	0.85	0.397	3.78	3	0.287	21	–	–	–	–	–	–	–	–	−0.23
Emotional symptoms																		
Parent	10	0.21	0.08, 0.32	3.43	0.001	29.60	9	0.001	70	0.22	0.07, 0.37	2.80	0.005	27.82	5	0.001	99	0.16
Teacher	6	0.14	−0.03, 0.30	1.62	0.106	14.22	5	0.014	94	0.15	−0.04, 0.34	1.49	0.135	12.76	3	0.005	98	−0.02
Self	4	0.19	−0.18, 0.40	0.74	0.456	59.58	3	0.001	95	–	–	–	–	–	–	–	–	0.05
Conduct problems																		
Parent	10	0.16	−0.03, 0.35	1.65	0.100	61.72	9	0.001	99	0.12	−14, 0.39	0.90	0.366	58.54	5	0.001	92	0.14
Teacher	6	0.22	0.10, 0.34	3.62	0.001	5.86	5	0.001	94	0.23	0.11, 0.34	3.76	0.001	3.29	3	0.349	8	0.06
Self	4	−0.25	−0.53, 0.03	1.74	0.082	6.66	3	0.001	96	–	–	–	–	–	–	–	–	−0.25
Hyperactivity																		
Parent	10	0.05	−0.06, 0.16	0.91	0.363	16.26	9	0.062	45	0.09	−0.02, 0.20	1.56	0.119	10.14	5	0.071	50	−0.07
Teacher	6	0.03	−0.16, 0.22	0.34	0.735	11.58	5	0.041	57	0.07	−0.12, 0.27	0.73	0.464	8.32	3	0.040	64	−0.17
Self	4	−0.21	−0.38, −0.04	2.44	0.015	1.09	3	0.780	0	–	–	–	–	–	–	–	–	−0.27
Peer problems																		
Parent	10	0.27	0.05, 0.49	2.40	0.016	72.71	9	0.001	88	0.28	−0.01, 0.58	1.90	0.057	69.83	5	0.001	93	0.22
Teacher	6	0.35	0.14, 0.57	3.23	0.001	19.09	5	0.002	74	0.22	0.07, 0.37	2.83	0.005	5.18	3	0.159	42	0.37
Self	4	0.41	0.24, 0.58	4.79	0.001	0.91	3	0.823	0	–	–	–	–	–	–	–	–	0.43
Prosocial behaviour																		
Parent	10	0.30	0.08, 0.52	2.61	0.009	107.41	9	0.001	92	0.24	−0.01, 0.49	1.87	0.062	84.62	5	0.001	85	0.25
Teacher	6	−0.10	−0.31, 0.12	0.88	0.376	17.14	5	0.004	71	−0.07	−0.34, 0.20	0.52	0.605	15.44	3	0.001	81	−0.15
Self	4	−0.00	−0.33, 0.33	0.01	0.990	6.82	3	0.078	56	–	–	–	–	–	–	–	–	0.04

^a^Mean of unweighted effect sizes—Hedge’s *g*

The results for Self-rated estimates have to be treated cautiously as these are based on just four studies. There is a pattern whereby the children and adolescents rate themselves as having fewer difficulties than those reported by Parents and Teachers. The exception is Peer Problems where the Self-rated score gives the highest effect size of all (0.41).

**Fig. 3:**
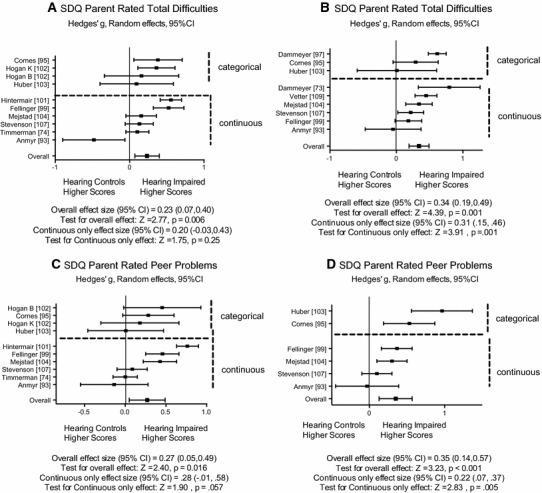
Forest plots for SDQ Total Difficulties and Peer Problems rated by Parents and by Teachers differentiating studies using categorical and continuous scoring. Effect sizes are Hedges’ *g* with 95 % confidence intervals estimated using a random effects model

Forest plots of the effect sizes for Parent and for Teacher Total Difficulties scores are presented in Fig. 3A and B. There are too few studies with self-rated scores to warrant presentation. A somewhat surprising finding is that of Anmyr et al. [93] which is the only study to produce a significant effect size that shows children with HI have lower Total Difficulties score than hearing controls, but this is found for Parent ratings only.

Only the SDQ Peer Problem sub-scale shows a consistent pattern of more difficulties for children with HI. The estimate is significant for Parent, Teacher and Self-rated scores (effect size of 0.27, 0.35 and 0.41, respectively). The effect sizes for Parent and Teacher scores for Peer Problems for individual studies are shown in Fig. 3C and D.

### Meta-regression for age

To test whether the effect size changed with age, meta-regressions were conducted for all scales for all raters. In 14 of these 18 regressions the effect size declined with age but only in the case of Self-ratings on the Emotional Symptoms scale did this reach significance (coefficient = −0.14, SE = 0.02, *P* < 0.02).

### Sensitivity analyses

The effect sizes presented above are based on both categorical and continuous measures of EBD. A sensitivity analysis was carried out on Parent and Teacher ratings to establish whether restricting the meta-analysis to continuous-only measures changed the pattern of results. This was not possible for Self-rated measures as there were too few values. The effect sizes based on continuous measures alone are presented in Table 5. The pattern of results remains largely unchanged.

The effect of removing the two studies where inclusion was uncertain was that the effect size for Parent Total Difficulties was little changed (0.25) and for Teacher Total Difficulties fell from 0.34 to 0.31 and in both cases the effect remained significant.

### Comparing the non-SDQ and SDQ study effect sizes

The methods of summarizing effect sizes described so far for the SDQ studies are different from those reported above for the non-SDQ studies. To allow a more appropriate comparison to be made, Table 5 includes in the final column a value for the unweighted mean effect size; the same summary as was given for the non-SDQ studies in Table 2. As reported above, the unweighted mean for the non-SDQ studies was 0.34 for Parent and 0.46 for Teacher ratings. For the SDQ studies, Total Difficulties means were somewhat lower at 0.20 for Parents and 0.31 for Teachers. This comparison must be treated with caution as the non-SDQ effect sizes are based on pooling of results from a wide variety of measures of EBD.

## Discussion

The analyses presented in this paper suggest that children and adolescents up to age 21 years with HI are likely to show an elevated rate of EBD. As rated on the SDQ by Parents and by Teachers (but not by the children themselves), these children and adolescents show an overall EBD score 0.23 of a SD above that of normally hearing children. The effect is more substantial for teacher ratings (0.34). There is an indication that this effect may decline with age but more studies (preferably longitudinal) are needed to adequately test for age-related changes. This pattern of a greater effect in teacher ratings is also found in the non-SDQ studies. In these studies, the effect sizes are slightly larger: namely 0.34 and 0.46 for Parent and Teacher ratings, respectively.

The findings from the SDQ studies suggest that Peer Problems showed the most marked and consistent increased risk of EBD. For Teacher ratings only, a significantly higher mean Conduct Problems score was obtained for children with HI. For Parent ratings only, there was a significant association between HI and elevated Emotional Symptoms. For all raters, there was no evidence that children with HI showed elevated levels of Hyperactivity. Parents report significantly less Prosocial Behaviour in children with HI.

The lower estimate of effect size in the SDQ studies might arise from a number of factors. First, the methods of measuring EBD might have an impact, e.g. in terms of the number and types of behaviour items included. Second, the adoption of a common large general population sample to provide an SDQ behaviour score for hearing children may also have reduced the effect size. In the non-SDQ studies, some of the control group scores are “super-normal”, i.e. unexpectedly low (e.g. [45]) and therefore might inflate the effect size estimate. Third, a number of the non-SDQ studies were published much earlier than the SDQ studies, all of which were published after the year 2000. Consequently, a smaller effect size may reflect improvements in the provision for psychological support for children with HI or changes in educational provision and policy which resulted in fewer difficulties being reported in the later studies. Children in these SDQ studies may also have benefited from early detection and confirmation of hearing loss as a result of universal newborn hearing screening. Since such screening and early confirmation are associated with better language [113] and better language is associated with reduced EBD [1], this could have led to a reduction in EBD in more recent studies. However, a direct test of the effect of early confirmation on later behaviour failed to detect a benefit [107] perhaps because the associated improvement in language was only to a rather low level, e.g. mean aggregate receptive language *z* score in children with early confirmed hearing impairments remained 1.76 SD below the mean score in the normally hearing comparison group.

The findings of Anmyr et al. [93] are a clear exception to the general pattern of the SDQ results. In that study the children with HI are rated as showing fewer EBD than controls. A distinctive feature of the sample in [93] was that all the children had received cochlear implants. The other study exclusively including children with cochlear implants was that by Huber and Kipman [103]. They too found no difference in parental ratings on the SDQ, although teachers in that study did report significantly more Peer Problems in the cochlear implant group compared to controls. The remainder of the SDQ studies had fewer than 33 % of cases receiving cochlear implants. It remains unclear whether those treated with cochlear implants may have fewer EBD or whether specific cultural factors such as parental expectations about behaviour may have resulted in the anomalous findings reported in [93], as these authors suggest.

### Hyperactivity

Our findings suggest no heightened risk for children with HI to present with symptoms of hyperactivity or inattention. This was somewhat surprising considering that there is some evidence for a possible link between HI and ADHD-type symptoms related to underlying cognitive abilities. Specifically, it has been shown that children with HI are more likely to have deficits in executive function and that scores on executive function were significantly related to Total Difficulties scores on the SDQ [81]. As attention and executive abilities are thought to be aspects of the underlying cognitive difficulties experienced by children with ADHD (see [114] and [115], respectively), it might be expected that children with HI would be likely to show a pattern of behaviour similar to ADHD.

However, evidence from studies using visually presented material to examine attention in HI participants is inconsistent. Tharpe et al. [45] found few differences between children with and without HI in visual attention performance. They suggested that the effects are influenced both by age and intelligence and differences between the groups disappeared when these factors were controlled statistically. By contrast, Mitchell and Quittner [38] reported significant attention deficits in children with HI on two of three attention tasks using the continuous performance test (CPT [116]). Performance on the CPT tasks was significantly associated with Parent and Teacher ratings of behaviour difficulties. However, the authors concluded that the attention problems in children with HI were not necessarily related to ADHD. Instead, they suggested that children with HI show a distinct pattern of impaired attention and problem behaviours. Furthermore, Quittner et al. [117] showed how the development of this pattern of impaired attention can be at least partially off-set by cochlear implantation. This was consistent with other evidence [118] which suggested that visual attention improves when children with HI are given cochlear implants.

There is no indication from the SDQ studies reviewed here that children with HI have a specific propensity to develop hyperactivity. Indeed the effect sizes for parent and teacher ratings are non-significant and for Self-ratings are in the opposite direction. This raises the question of why overviews of the earlier studies on children with HI have concluded that hyperactivity was a particularly salient feature of their behaviour.

There is a possibility that the SDQ is a less valid or less sensitive measure of hyperactivity or ADHD as it only relies on five items to assess this pattern of behaviour. However, studies of large general population samples in Europe [119] and in the USA [120] and of clinical samples of children with ADHD [121, 122] have supported the validity of the SDQ as a measure of hyperactivity. A summary of such data is given in a meta-analysis of 48 studies (*N* = 131,223) on the psychometric properties of the SDQ [123].

It appears then that earlier studies of ADHD-related behaviours in children with HI may have over-estimated such an association possibly as a result of the difficulties that have been noted in the assessment of such behaviour in deaf children, such as failure to follow directions being the consequence of hearing impairment rather than inattention [124].

### Mediating and risk factors

It is important to note that the present meta-analysis does not directly examine the possible factors mediating the impact of HI on EBD but these need to be considered in interpreting the findings. It has been suggested that low non-verbal IQ [3, 45] and language impairment may be crucial mediators [5, 6]. The difficulties in language acquisition experienced by children with HI may contribute to the risk of EBD in a number of ways. The first is via a failure to effectively understand or communicate information about needs and wants with others. The second is via deficits in emotional and behavioural regulation that are, in part, dependent on language processing. These are interpersonal and intrapersonal processes respectively and children with HI may have deficits in either or both of these with consequently adverse effects on behaviour [14]. It has also been suggested that the language deficits in children with HI are related to intrapersonal cognitive processes such as attention [125] and executive function [126] that in turn create a vulnerability to poor behavioural regulation.

The optimal method for detecting risk factors, i.e. features that make a child with HI more likely to develop EBD, is via a multiple regression moderator analysis based on the child as the unit of analysis [127]. The present meta-analysis only has the study as the unit of analysis and using a meta-regression tested whether age was a moderator of the effect of HI on EBD. It was not. The approach adopted by Theunissen [4] was to survey whether specific risk and protective factors had been found to relate to EBD within HI samples. Using this approach, age at detection and intervention of hearing loss, the presence of additional disabilities, communication skills, intelligence, type of school, and number of siblings were suggested as possible influences on EBD in children and adolescents with HI.

### Peer problems

The review of the studies using the SDQ identifies Peer Problems as the area with the most difficulties for children with HI. Other studies reinforce this notion of peer relationships and friendships as being problematic for children with HI. Henggeler et al. [128] found that the mothers and the fathers of adolescents with HI, but not the adolescents themselves, rated their relationship with friends as showing more aggression than did the parents of hearing adolescents. Parents reported that their deaf children were socially isolated and did not participate in structured activities [2]. This effect was found for otherwise well-functioning children and not just for those with behaviour problems. A school-based study also reported that although not rejected by others, deaf pupils in mainstream schools may feel isolated [129]. As a result, they show lower self-esteem in relation to peer relationships [130]. One factor likely to be contributing to peer relationship difficulties is the delay in acquiring pragmatic language skills shown by children with HI [131].

There is some evidence that social skill training can have an enduring beneficial effect on peer relationships in deaf children [132]. Replication and extension of these findings on intervention are clearly needed as the findings in this review suggest that peer relationship problems may be a real risk for children with HI. Further research is necessary to illuminate the processes by which children with HI become more socially isolated; in particular into the role, difficulties in language ability are likely to play as a mediator in this association. The clinical and service implications of social and mental health problems in deaf children and adults have been recently reviewed [133].

### Limitations on the study

As discussed above a major feature of the studies being reviewed was their heterogeneity in terms of factors such as age, severity of hearing loss, numbers of children with cochlear implants and types of control/comparison groups employed. There were also a wide range of methods used to measure EBD. This in part was addressed by separating studies applying the SDQ from those using other questionnaires. It is feature of all the studies reported here that they were reliant on partner, teacher or self-ratings on questionnaires to obtain assessment of EBD. It is uncertain what affect the absence of more clinically sensitive methods this might have on the level of EBD found in children with HI [134].

## Conclusions

The analyses presented here confirm that children with HI are more likely to show EBD than other children. The effect may be less marked than earlier studies suggested. The ratings of EBD by teachers show the largest effect. There is no indication that HI is related to hyperactivity or ADHD-related behaviours. It is in the area of peer relationships that the most marked behaviour difficulties for children with HI are found.
